# Legumes and common beans in sustainable diets: nutritional quality, environmental benefits, spread and use in food preparations

**DOI:** 10.3389/fnut.2024.1385232

**Published:** 2024-05-06

**Authors:** Silvia Lisciani, Stefania Marconi, Cinzia Le Donne, Emanuela Camilli, Altero Aguzzi, Paolo Gabrielli, Loretta Gambelli, Karl Kunert, Diana Marais, Barend Juan Vorster, Katherine Alvarado-Ramos, Emmanuelle Reboul, Eleonora Cominelli, Chiara Preite, Francesca Sparvoli, Alessia Losa, Tea Sala, Anna-Maria Botha, Marika Ferrari

**Affiliations:** ^1^Research Centre for Food and Nutrition, Council for Agricultural Research and Economics, Rome, Italy; ^2^Department of Plant and Soil Sciences, Faculty of Natural and Agricultural Sciences, University of Pretoria, Pretoria, South Africa; ^3^Aix-Marseille University, INRAE, INSERM, C2VN, Marseille, France; ^4^Institute of Agricultural Biology and Biotechnology, National Research Council (CNR), Milan, Italy; ^5^Research Centre for Genomics and Bioinformatics, Council for Agricultural and Economics Research, Montanaso Lombardo, Italy; ^6^Department of Genetics, Stellenbosch University, Stellenbosch, South Africa

**Keywords:** sustainable diets, legumes, common beans, traditional recipes, plant-based diets

## Abstract

In recent decades, scarcity of available resources, population growth and the widening in the consumption of processed foods and of animal origin have made the current food system unsustainable. High-income countries have shifted towards food consumption patterns which is causing an increasingly process of environmental degradation and depletion of natural resources, with the increased incidence of malnutrition due to excess (obesity and non-communicable disease) and due to chronic food deprivation. An urgent challenge is, therefore, to move towards more healthy and sustainable eating choices and reorientating food production and distribution to obtain a human and planetary health benefit. In this regard, legumes represent a less expensive source of nutrients for low-income countries, and a sustainable healthier option than animal-based proteins in developed countries. Although legumes are the basis of many traditional dishes worldwide, and in recent years they have also been used in the formulation of new food products, their consumption is still scarce. Common beans, which are among the most consumed pulses worldwide, have been the focus of many studies to boost their nutritional properties, to find strategies to facilitate cultivation under biotic/abiotic stress, to increase yield, reduce antinutrients contents and rise the micronutrient level. The versatility of beans could be the key for the increase of their consumption, as it allows to include them in a vast range of food preparations, to create new formulations and to reinvent traditional legume-based recipes with optimal nutritional healthy characteristics.

## Graphical abstract

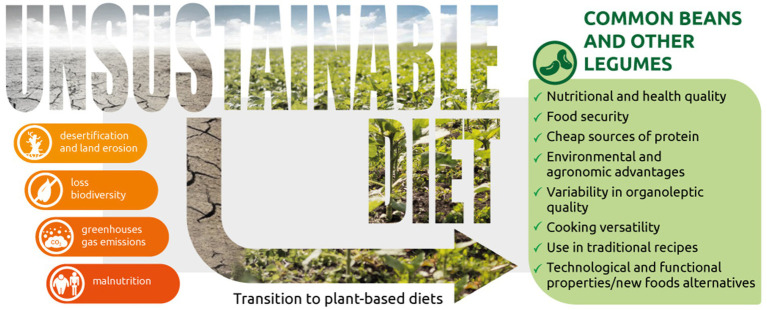

## 1 Introduction

In recent years, with the lengthening and diversification of the food chain, refined foods rich in fats are easily accessible due to the increase of industrial processing and transport over long distances (1). This has led to a shift in the way of local and seasonal foods, especially fiber-rich plant foods, as well as an increase in energy-dense processed foods high in refined starches, sugars, saturated fats, and salt (1, 2). High-income countries have moved towards such energy-dense and animal-based food consumption patterns resulting from an intensification of food production. This is causing significant environmental damage with an increasingly rapid process of environmental degradation and depletion of natural resources (3, 4). Although abundant food is produced for all humans, the coexistence of extreme and opposite forms of malnutrition still globally exists. If in one part of the world there is a high incidence of malnutrition due to excess, obesity, and non-communicable diseases, in the other there is an increasing number of people afflicted by chronic food deprivation (5). Many people suffer from what is called “hidden hunger”; malnutrition due to a lack of micronutrients which prevents them from leading a healthy life (6, 7). The increase scarcity of available resources (especially water and soil), in combination with the demographic increase in world population and an increase in the consumption of processed foods and other products of animal origin, make the current food system unsustainable (8). The adoption of a sustainable, therefore, diet seems to represent an ideal tool through which to reform the global food system and stands as one of the most significant challenges of humanity (9–11).

Changing eating habits and consequently the food supply towards sustainable models, and reorienting food production and distribution might create significant human and planetary health benefits. The urgency of the transition towards a sustainable diet has recently been highlighted by a publication edited by the Eat Lancet Commission (11). The Commission proposes to change the food policies of the world by directing them towards a diet with a strong plant component. According to the report, this shift is uniquely sustainable both from a nutritional and environmental point of view.

Many studies have further shown that substituting protein-rich plant food for meat is beneficial from both an environmental animal welfare and human health point of view (12–17). The consumption of plant-based proteins from legumes, such as common beans, is increasing on a global basis, and represent a sustainable, and healthier option than animal-based proteins (18). Therefore, in plant-based diet formulations, it is necessary to insert sources of high quality and quantity of protein and major micronutrients (19). Legumes including common beans are an economic source of nutrients as well as a potential source of income, especially in developing and emerging countries, where access to proteins of animal origin is often lacking and represents a serious nutritional issue (20–23). In addition to the problem of insufficient protein and energy intake, deficiencies in micronutrients such as iron, iodine and vitamin A affect millions of people in poor and middle-income countries, including Africa (24). In these populations’ legumes, especially common beans, can represent a source of vitamins, iron, zinc and biologically active phytochemicals (25–27).

Due to their nutritional and tecno-functional properties the importance of legumes is not limited to low-income countries, and their consumption has increased in developed countries for general and specific groups of the population (28, 29). This is mainly a consequence of two phenomena: the growth of vegetarianism and the demand for protein not derived from wheat or other gluten-containing grains (30, 31). However, the cultivation and consumption of legumes is still negligible as it is hindered by many nutritional, organoleptic and socio-economic barriers (32). It is, therefore, necessary to raise the awareness of the benefits of legumes from a nutritional and environmental point of view, emphasizing their potential role as a food, as well as an ingredient in traditional and non-traditional recipes and in the formulation of new products. The purpose of this review has been to highlight recent knowledge on the role of legumes and common beans in a sustainable diet. A particular focus is on common beans as a key food component in numerous recipes. The review is be based on the analysis of healthy, environmental, socio-economic elements that prevent or facilitate the consumption of legumes including the means used to reduce anti-nutritional components, ranging from simple home preparation methods to modern breeding technologies. Always with a view to combining the traditional and current aspects of such a versatile food, we investigated the food uses that see them as protagonists in numerous traditional dishes all over the world and their use as ingredients in preparations for the new food market.

## 2 Benefits of legumes

### 2.1 Nutritional composition and health benefits

Although the composition of legumes depend on several factors, such as the species, variety, environmental factors, and the cooking method applied, their nutritional profile is remarkable and provides many benefits (Table 1). Legumes are an excellent source of B-group vitamins, such as folate, thiamine and riboflavin, and vitamin C (23). Minerals, including potassium, calcium, magnesium, zinc, copper and iron are present in legumes in high amounts. In contrast, legumes are low in sodium, and this is desirable considering the recent trends encouraging salt reduction (28, 53). Furthermore, legumes are rich in linoleic and oleic acid, and bioactive compounds which have functional beneficial properties (33, 34).

**Table 1:** Summary of original research articles related to the nutritional and environmental benefits of legumes, their use and preparation.

*Nutritional composition and health benefits of LEGUMES*		
*Author*	*Year*	*Outcomes*
Celmeli et al. (28)	2018	Different common beans landraces had significative differences in the content of protein, Se and Zn.
Masum-Akond et al. (33)	2011	Different common bean genotypes contained different amount of minerals and phytic acid.
Kibar and Kibar (34)	2019	Nutritional composition and bioactive profile in common beans changes after storage.
Nosworthy et al. (35)	2017	Legumes, except whole green lentils and split green peas, would qualify as sources of protein with protein ratings between 20 and 30%.
Bonke et al. (36)	2020	Legumes have low amount of lysine, except for lentils.
Xu et al. (37)	2012	Different legumes varied in phytochemical and antioxidant profile.
Felix-Medina et al. (38)	2021	Incorporation of legume as a food ingredient contribute to increase the nutritional profile of snacks.
Chen et al. (39)	2020	*In vitro* digestion of legume fiber produced short chain fatty acids.
Bassinello et al. (40)	2020	Incorporation of bean flour in a cake improved protein digestibility, total dietary fiber, and raised Fe and Zn contents.
Serra-Mayem et al. (41)	2020	New mediterranean diet model suggests the daily legumes consumption.
*Agronomical and environmental benefits of LEGUMES*		
Nassary et al. (42)	2020	Rotation of maize and common bean could improve crop yields.
Rekling et al. (43)	2016	Cultivation of legumes lead to economic competitive cropping systems and positive environmental impacts.
Del Borghi et al. (44)	2018	Legumes packaging and crop cultivation account for major environmental problem.
Tidåker et al. (45)	2021	Processing, packaging and transport affect the environmental impact of pulses.
Bandekar et al. (46)	2022	Cooking process is the primary contributor to the global environmental impact of legumes.
*LEGUMES in traditional recipes and innovative preparation*		
Didinger et al. (47)	2023	Creation of “Bean Cuisine” with 56 recipes bean-based promoted the consumption of legumes among the participants.
Chiang et al. (48)	2020	Creation of indicator of cuisines’ sustainability.
Han et al. (49)	1999	Mixture of cereals and pulses increases the protein quality determined by DIAAS.
Ziarno et al. (50)	2020	Bean-based beverages obtained from the germinated seeds of white bean change the fatty acids profile of pulse.
Laleg et al. (51)	2016	Legume pasta showed a low glycemic index and high nutritional quality.
Arribas et al. (30)	2020	The addition of high percentages of bean improved the bioactive compound content of pasta.
Bassinello et al. (40)	2020	Incorporation of bean flour in a cake improved protein digestibility, total dietary fiber, and raised Fe, and Zn contents.
Natabirwa et al. (52)	2020	Bean-based snack formulation exhibited desirable nutritional and sensory properties.
Silva et al. (31)	2021	Gluten free rice and beans biscuits presented good protein mineral and fiber contents.

Legumes are further an excellent source of protein (20–45% on weight) and represent important plant-based sources of this macronutrient (23, 35). Protein quality in legumes is, however, limited by the low concentration of the essential sulphur containing amino acids: methionine, cystine and cysteine, as well as tryptophan (53–55). This weakness can be supplemented by combining legumes with grains, that introduce great amounts of sulphur containing amino acids (55). Conversely, many grains are particularly low in lysine, and the most notable category of plant-based ingredient that can complement this lack is legumes that have a high lysine content, approaching the daily recommended intake provided by about in just 100 g of lentils or peas (36).

**Figure 1:**
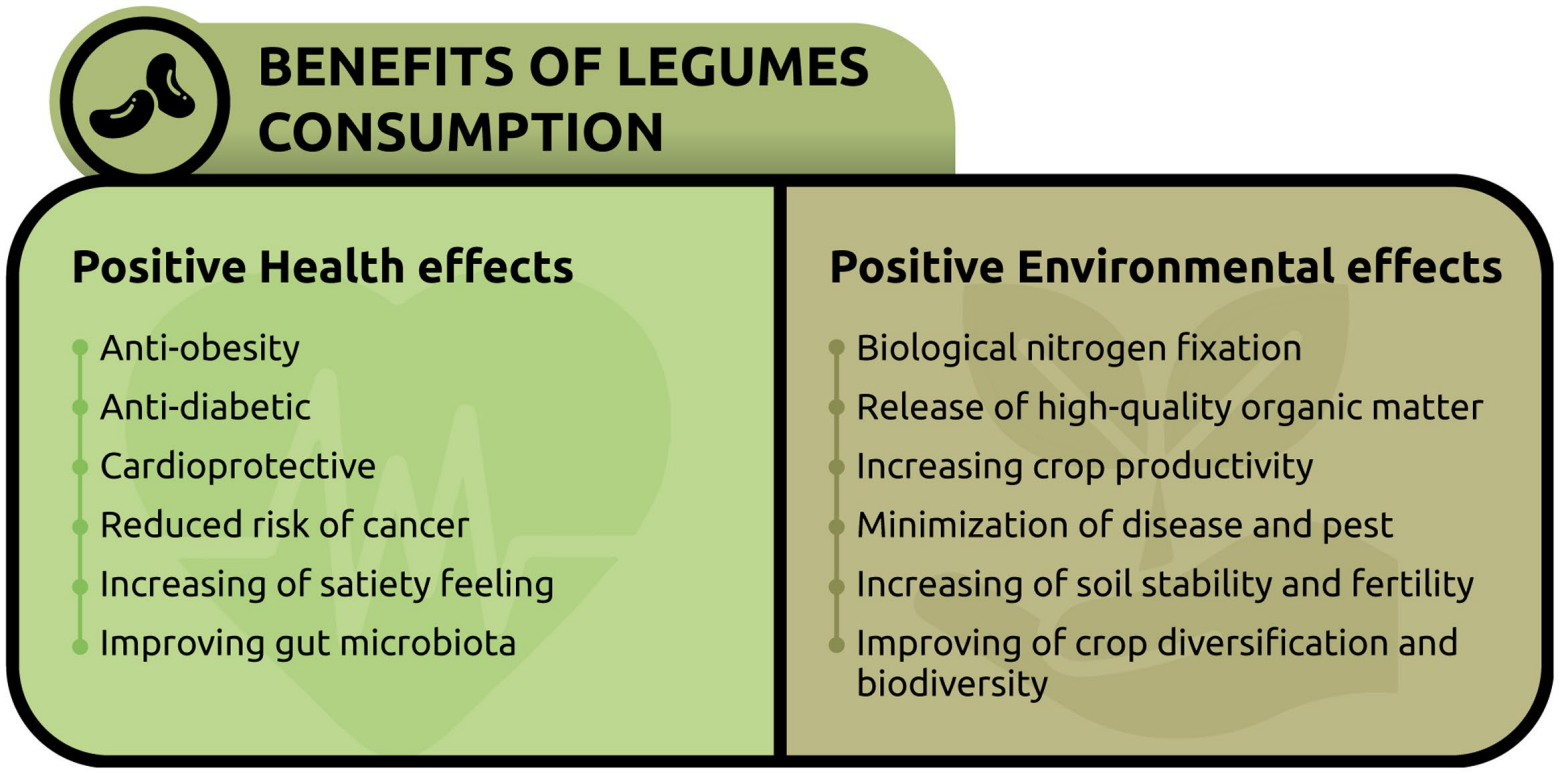
Nutritional, agronomical and environmental benefits of legumes.

Scientific evidence also supports the health benefits of consuming a plant-based diet and increasing the intake of legumes thanks to their nutritional characteristics (Table 1) (35). Legumes, if consumed on a regular basis, contribute to reduced risk of mortality because of their benefits against major chronic diseases: obesity, diabetes, cardiovascular diseases, and some types of cancer (37, 38, 53, 55, 56). Legumes generally can reduce cardiovascular disease risk via improvements in blood pressure, lipid profile, inflammation, blood sugar metabolism and body weight, and offer a food-based solution to decreasing risk of developing type 2 diabetes, as reported in Figure 1. Indeed, in diabetic patients’ diets, legumes help to moderate blood sugar levels after meals, improve insulin sensitivity and glycaemic control (39, 53, 57).

Part of the health benefits of legumes may also be attributed to the low amount of fat, and to the presence of different complex carbohydrates: resistant starch, hemicellulose, oligosaccharides, lignin, and dietary soluble fibre (31, 55). The non-digestible carbohydrates of legumes can pass unchanged through the stomach and small intestine until they reach the colon, where they act as “prebiotics” for the beneficial bacteria that reside there (39). The bacterial fermentation leads to the formation of short-chain fatty acids (SCFA) which can improve colon health by promoting a beneficial gut microbiome and reducing the risk of colon cancer (39, 40, 57, 58). Furthermore, slowly digestible carbohydrates, proteins and fibres from legumes can increase the feeling of satiety and therefore reduce the risk of obesity (59) (Figure 1). Data from the National Health and Nutrition Examination Survey (NHANES), highlights that adults who consumed a variety of legumes had significantly lower body weights compared with those who did not consume legumes (57).

Legumes are further an integral part of many healthy eating patterns, including the Mediterranean style of eating (57). Serra-Mayem et al. (41) in the revision of the Mediterranean food pyramid, which incorporated recent discoveries on the sustainability and environmental impact of this dietary model, recommended the daily consumption of one small legume serving. The daily consumption of legumes has also been recommended for other types of diets such as the DASH eating plan, vegetarian and vegan diets, lower glycaemic-index diets, and diets for celiac (40, 57).

The emphasis on increased consumption of food legumes is also reflected in government-issued dietary guidelines (58). With the FAO’s assistance, about 100 countries have developed food-based dietary guidelines (59). According to an evaluation of these guidelines (60) about 87% of them recommend regular inclusion of pulses in the diet, but in general terms without specifically pointing out their nutritional value or health benefits. The guidelines of about 27% of the world’s countries mention that pulses are important sources of protein as animal foods, talking about the health benefits of reducing the consumption of meats and substituting it with pulses. However, only 15% of national dietary guidelines refer to the high iron content of pulses; and 20% point to the fact that they contain high dietary fibers. In just 8% of the guidelines, health benefits like management of obesity and diabetes are discussed.

### 2.2 Agronomical and environmental benefits

Legumes, thanks to rhizobia (nitrogen-fixing bacteria with which they live in symbiosis), can use fixed nitrogen and produce amino acids from ammonia. This is the reason why they tend to have a higher amount of protein than other plant families (61). From an agroecological point of view, legumes play an essential role in the cultivation of sustainable agriculture also through the release of high-quality organic matter into the soil, and the consequent increase in its productivity, of both intercropped crops and crops to be cultivated subsequently (Figure 1) (62–66). Intercropping and rotation of legumes with grains, or other non-legume crops, is one of the most important elements of sustainable intensification in densely populated areas, due to benefits such as increased yield, higher nitrogen use efficiency, minimization of diseases and pests, improved access to other essential elements such as phosphorus which expand soil fertility (42, 67, 68). Grains grown in rotation after pulses yield on average 1.5 tons more per hectare than those grown without pulses, equivalent to the effect of 100 kilograms of nitrogen fertilizer (62). Furthermore, increasing the organic carbon status of the soil, legumes consequently allow to reduce the use of external fertilizers and the release of greenhouse gases in the form of CO_2_ and N_2_O associated with them (63, 66, 68). In fact, the production and application of synthetic fertilizers in the agricultural production system are the major contributors to GHGs emissions; and N fertilization contributes 36–52% of total emissions. A confirmed by Rekling et al. (43) integrating legumes into cropping systems, rotation can reduce nitrous oxide emissions by 18 and 33% and N fertilizer use by 24 and 38% in arable and forage systems, respectively, compared to systems without legumes (Table 1). Legumes in the cropping system also improve soil physical conditions, such as aggregate stability and soil structure, while reducing bulk density and facilitating nutrient circulation by promoting water retention (Figure 1) (67, 69). Finally, thanks to the high genetic variability of legumes, it is possible to develop high-yielding, climate-resistant varieties and, through crop rotation with legumes, increase crop diversification and agrobiodiversity (70). For example, legumes enhance earthworm activity, which along with the root channel of pulses, increases soil porosity, promotes aeration, increases water-holding capacity, and percolates deeper into the subsoil (67, 69–71).

Processing, including cooking, and packaging of legumes are important steps in the supply chain that need to be investigated since they can have a significant environmental impact (44). As reported in Table 1, an interesting Swedish study (45) compared the environmental impact of domestic and imported pulses. Authors found that contribution from processing and packaging is different between pulses purchased either dry and then cooked at home and the canned one. For canned legumes, the energy use related to retorting was almost negligible when compared with energy use in production and waste management of the packaging. For pulses purchased dry and cooked at home, the energy use was 3–6 times higher than for production of the packaging. Bandekar et al. (46) conducted a Life Cycle Assessment (LCA) for different variety of legumes considering the stages of production and consumption, including cooking, and they discovered that the consumer stage dominated the environmental impacts of pulses for all varieties and scenarios. Electricity consumed during cooking was the principal driving factor for cradle-to-grave impact of pulses and for consumer stage. The authors concluded that cooking large batches of legumes and then refrigerated, could greatly reduce environmentally impact in terms of global warming potential, fossil resource scarcity, water consumption, freshwater eutrophication, and marine eutrophication.

### 2.3 Production and spread of legumes

The concept of food has also changed several times with the progress of civilization, the expansion of knowledge, the industrial revolution, and emergence of new processing and preservation techniques. In this regard, legumes have been a fundamental part of the human diet due to the advent of agriculture and the development of civilization in the Middle East, Asia, Americas and Europe particularly in Mediterranean cultures (72, 73). Since the 1950s, there has been, however, a decline in the production of legumes, due to a series of factors including the transition of the population to dietary patterns characterized by increased consumption of meat (67, 72, 74). With the rise in large-scale poultry, livestock and aquaculture production, there has been also a relative decline in the prices of foods originating from animals. This is consistent with the drop in consumption of pulses in the diets of most regions of the world (60).

The global area and total legume production increased steadily during the 2000s, especially during the 2001s (67). Nowadays, legumes share an area of around 81 million ha with production of more than 92 million tons globally (75). The global food supplies through pulses remained, however, negligible and amounts to merely ∼1.0% of the total food supply, and 1.2% of the vegan food system (67, 74). The main socio-economic limitations to legume production are the relatively low yields compared to grains, the sensitivity to biotic and abiotic stresses, and their cultivation in harsh environments (76, 77). Legume production, likewise, has been hampered by lack of varietal protection, limited availability of genomic resources (78), and unavailability of seeds suited to various environments as well as lack of knowledge of different types of legumes by farmers and governments in agricultural policy planning (74, 76, 78). There are also other constraints to the spread of legume cultivation, including lack of adequate production markets, especially considering post-harvest losses and costs (76). Such difficulties occur mainly in high-income countries, where agricultural practices and their business models dependent on high-yield intensive systems. This hinders farmers from producing legumes, because it is economically unsustainable and socially undesirable (79). It is precisely in high-income countries that there has also been a reduction in the consumption of legumes in recent years.

#### 2.3.1 Barriers to legumes consumption

The observed decline in legumes consumption was attributed to factors including the lack of clarity regarding the quantity and serving size of beans and other legume products despite the availability of national dietary guidelines (80). Among the causes of the low consumption of legumes there is the consumers’ lack of knowledge of the nutritional and health value and the perception that they are an unappetizing and “unattractive” food (73). These considerations are mainly attributable to the digestive effects linked to their consumption: the formation of SCFAs by the human gut microbiome is associated with the production of intestinal gas, composed of hydrogen, carbon dioxide and methane, that in addition to causing flatulence and bloating, can be associated with diarrhea and abdominal pain (58).

Moreover, several types of anti-nutritional factors with toxic potential-related problems and harmful effects to human health, have been measured in legumes. These factors include phytic acid, enzyme inhibitors, saponins, phenolic compounds, lectins and haemagglutinins (33). Anti-nutrients are natural constituents of plant foods with different functions, that can interfere with the nutritional value of legumes, by reducing mineral absorption, protein digestibility and causing toxicity and health disorders when present in high concentrations. For example, legume seed proteins have reduced digestibility, hindered by the protein structure and to the presence of trypsin inhibitors, phytates, tannins and lectins (61, 81).

It is therefore clear that the study and application of food processing technologies to reduce the content of anti-nutrients in legumes represents one of the main strategies to stimulate their consumption. This should also be accompanied by political, commercial and communication strategies to promote the health and environmental characteristics of the legumes (58).

In this perspective, the mismatch between the current reality and the different potential benefits of legume production and consumption has therefore initiated a wide range of research, development and marketing activities have been established to examine the agronomic applications of legumes, to close yield gaps, and to assess how they might be able to improve the environmental profile of farming and contribute to the creation of a healthy and sustainable food system (79). Due to climate change, which has become a global challenge since the second half of the 20th century, in fact the pressure to re-consider the role of agriculture and food chains has also increased. This includes with supporting farmers to improve agricultural productivity to ensure a stable supply of affordable food (82, 83).

## 3 Legumes in traditional recipes and innovative preparation

The use of legumes for the preparation of local and traditional dishes represents an excellent sustainable and healthy strategy to encourage their consumption by different populations, making them key foods of Sustainable cuisine (Table 1) (47). Sustainable cuisine combines several factor: type or variety of products, method of growing or raising those products, knowing when and how the food product is harvested, slaughtered or caught and how it is packaged and delivered (48). Sustainable cuisine is thereby a system that uses food products grown, harvested, processed, packaged, and shipped or distributed with minimal environmental, economic and social impact (48, 84).

In recent years, there has been an increase in interest in traditional and local foods which represents an important sector in the global food economy (85). Traditional foods products are usually associated with quality, safety, and with local tradition (85, 86). They are often prepared from fruits, vegetables, meat, fish, milk, eggs, nuts, legumes, and seeds (86). In many societies, traditional diets contain both cereals and legumes which are complementary for most amino acids and may meet protein requirements for adults (49). Therefore, local recipes which have legumes and grains as main ingredients, are world-wide applied: *dhal with rice* in India, *beans with corn tortillas* in Mexico, *tofu with rice* in Asia, *sorghum and cowpeas* in Africa, Bambara groundnut and maize kernels in Zimbabwe, or *rice and beans* in Southern Africa and Latin America (61). In China, the staple food commonly consumed are the *eight-treasure porridge* (about eight kinds of grains and pulses cooked together), *millet with mung beans*, and *adlay with adzuki beans* (49). Conversely, most African diets mainly include grains as a staple, which provide significant amounts of energy and protein, but limit the intake of essential amino acids, so the combination of grains with legumes can represent a vital solution to malnutrition in Africa (7). Furthermore, the combination of legume-based ingredients with different products can potentially expand the utilization of legumes beyond traditional uses and consumption patterns, and there is great interest in the food industry for the use of legumes in various food systems (58).

The nutritional and functional properties of legumes as well as their versatility enhance their culinary value, and the possibility to use them to prepare several recipes, even replacing other less sustainable ingredients. For example, legumes could be a great candidate for egg replacement (87): legume protein isolates and concentrates offer similar functionality to egg, such as binding, foam stabilization, emulsification, gelling and humectancy (87, 88). Similarly, in many cuisines of the world, pulses that mimic the consistency of dairy products have long been used to produce drinks or other products, such as tofu and tempeh. They have recently entered the Western market as alternative protein products to those of animal origin (89).

Nowadays, modern technologies can be used to obtain milk substitutes. They can be obtained by subjecting seeds to biological processes such as germination and fermentation by an appropriate lactic acid bacteria strain (50). Legumes are in fact a good substrate for microbial fermentation, which can improve the digestibility of proteins ensuring a product with good sensory characteristics, and suitable for lactose-intolerance (Table 1) (89). The western market of traditional products has adapted to this scenario, as happened with pasta, typical of Italian cuisine, but present throughout the world. Wheat, in fact, can be replaced by other starchy matrixes. Recently, the diffusion of pasta composed exclusively of different types of legume flours has increased, resulting in a gluten-free product with low glycaemic index and high nutritional quality (30, 51).

Legume flour can also be useful for bakery food (Table 1). Bassinello et al. (40) developed a new kind of cake by replacing wheat flour with that of rice flour, corn starch and extruded split bean flour. This new food product, when analysed for their nutritional characteristics, clearly showed that the baked cakes are a protein source with a satisfactorily digestibility of starches and proteins and with little amounts of anti-nutritional factors. Natabirwa et al. (52) further developed a nutrient-rich extruded bean-based snack, containing 82% beans, 10% maize, 5% orange-fleshed sweet potato, and 3% amaranth. The snack revealed desirable nutritional and acceptability properties. Bean flour considerably increased the protein, iron, and zinc content of the snack, when compared to extruded corn products (52). In a similar way, Silva et al. (31) developed biscuits by combining rice and beans in a single preparation, inspired by the classic Brazilian staple food rice and beans. The different formulations were subjected to instrumental, physical, nutritional, and sensory tests. The nutritional composition compared with “control” biscuits, containing only wheat flour. Since rice and bean formulations have a sufficient amount of protein, mineral and fiber contents, these formulations represent a possible option for inclusion into gluten-free diets.

## 4 Common beans as a key legume

Common beans (*Phaseolus vulgaris* L.) are the third most important legume crop grown worldwide, after soybeans and peanuts (88), and dried beans are the most produced in developing countries (42, 60). Sub-Saharan Africa will further account for most of the growth of the world’s population over the coming decades, increasing the necessity for a sustainable provision of nutritious food. Common bean has an important role to play in this regard. It is thus likely to become an increasingly significant food source in Africa where it will represent an important legume supporting nutritional security (90–92).

### 4.1 Spread and use of common beans

Common beans feed more than 300 million people linked to agricultural economies across the world (93). Common beans are mostly sold as dry beans and a small proportion as fresh pods (60). Dry bean production has increased by about 60% since 1990 to 2020, and the area harvested increased by 36% in the same period (60, 93). Regionally, Asia leads in dry bean production with about 43% of global production, followed by America (29%), and Africa (26%) (93). All common bean lines grown in the different continents are the result of a process of domestication and evolution from wild forms (*Phaseolus vulgaris* var. *aborigines* and *Phaseolus vulgaris* var. *mexicanus*) found exclusively in the Americas (94). Beans are a rich global resource of biodiversity thanks mainly to landraces that provide genetic diversity across a wide range of seed types, produced according to diverse cultural practices (93). For centuries, farmers have maintained their varieties and have exchanged their seeds with surrounding areas, mainly in local markets (54). It is, however, not always easy to know the use and name given by farmers to their old landraces. and beans have probably been selected under dissimilar criteria and pressure (54). While landraces have a high value to preserve genetic variability and possibly organoleptic qualities, the local production cannot satisfy the current demand of the food industry. Despite this, in different countries common bean landraces are still grown, especially due to their strong link with rural gastronomy (80). Among the main food crops, the common bean also shows large variations in terms of cultivation methods, growth habit - in different environments and altitudes - as well as, in terms of plant physiology and architecture, relative duration of the reproductive cycle, and maturation time (95, 96). For these reasons, common beans show a wide range of size, shape, color and maturation time, tenderness and cooking quality of the edible plant parts (54, 57, 94).

Common beans are further greatly polymorphic with several ethnic varieties existing and with characteristics and names specific to different areas, regions, and or locality (9, 96). Consumers of different countries and regions show specific preferences for various combinations of seed characteristics, cooking time, and storability. Bean classification into “commercial types” is, therefore, also used which is related to market preferences (54). According to Doma et al. (97) the main characteristics that push consumers to choose beans are nutritional value, taste/consistency and versatility in cooking. While the strongest barriers to the bean’s consumption are: (I) beans are not part of traditional diet, (II) flatulence/abdominal discomfort, (III) knowledge gap about preparation and how to include them in daily diets (58, 83, 97), (IV) changes in lifestyles and less time available for cooking, and (V) greater availability of affordable processed foods easily to prepare (98). In this regard, consumers in developed countries avoid home-cooking beans due to the long cooking time involved. By contrast, consumers in Asia, Africa, and South America typically cook legumes at home, but choosing those that are relatively easy to cook, with shorter cook time (lentils, mung bean, and black gram) (97). In fact, long cooking time is a factor that constrains the consumption of pulses; although this phenomenon can be lessened by soaking, pressure cooking and the availability in many countries of pre-cooked dishes and ready-to-eat canned legumes (60).

### 4.2 Nutritional characteristics of common beans

Common beans are a rich and a relatively inexpensive source of proteins for a large part of the world’s population, mainly in developing countries, containing from 17 to 31% protein on a dry weight basis, in accordance with its variety (99). Common beans are further used as one of the cheapest protein sources and considered as the poor man’s meat (34, 99). Compared with animal sources of protein, beans have only 4% fat, and are also an excellent source of dietary fibre, vitamins, and minerals (Table 2) (25, 33, 100). Among carbohydrates, beans also contain oligosaccharides, mainly raffinose, which have been reported to possess prebiotic properties (23, 53).

**Table 2:** Summary of original research articles related to the nutritional benefits of common beans, strategies for improving their nutritional quality, and their spread and use.

*Nutritional composition and health benefits of COMMON BEANS*		
*Author*	*Year*	*Outcomes*
Landa-Habana et al. (25)	2004	Total starch was higher in common beans cooked with the traditional procedure than in the autoclaved samples, and did not change during storage at 4°C.
Masum-Akond et al. (33)	2011	Different common bean genotypes contained different amount of minerals and phytic acid.
Kibar and Kibar (34)	2019	Nutritional composition and bioactive profile in common beans changed after storage.
Alcazar-Valle et al. (100)	2020	Four different species of *Phaseolus* showed significant differences in the content of phenolic compounds and antioxidant potential.
Oomah et al. (101)	2014	Heat treatment of common beans had variable effects on the phenolic composition of hulls without significantly altering the antioxidant activity in black and pinto bean hulls.
Rodriguez Madrera et al. (102)	2021	The phenolic compounds in common beans with colored coating showed greater antioxidant capacity than those with white coating.
Carbas et al. (103)	2020	Navy beans and pink-eyed peas have shown higher protein and amino acid content. Red kidney beans, cranberry, and yellow Arikara beans have the highest content of phenolic compounds.
Blair et al. (104)	2013	The most important gene for seed coat Fe in common beans was on linkage group B04.
Gashu et al. (105)	2021	There is geospatial variation in the composition of micronutrients of staple cereal grains for most of the cereal production in Ethiopia and Malawi.
Kinyanjui et al. (106)	2015	Dehulling, soaking in high pH and salt solutions reduced the cooking time of different bean varieties.
Lv et al. (107)	2015	The bioavailability of Fe from phytoferritin is higher than animal ferritin, suggesting that the binding affinity between plant ferritin and receptors may be greater than that of animal ferritin.
Shi et al. (108)	2018	In different Canadian legumes, soaking decreased lectins and oxalates but not phytic acid. Cooking reduced all factors except phytic acid in beans and soybean.
Sparvoli et al. (109)	2021	Biofortified food products with common bean flour devoid of active lectins and with reduced phytic acid lost the haemagglutinating activity, increased α-amylase inhibitory activity and iron bioavailability.
Antoine et al. (110)	2021	In different meals, chickpeas reduced vitamin D and mineral transfer to the aqueous phase during digestion. The presence of meat induced a decrease in vitamin D stability.
*Strategies for improving nutritional quality and spread and use of COMMON BEANS*		
Didinger et al. (47)	2023	Creation of “Bean Cuisine” with 56 recipes bean based which contributed to promoting the consumption of legumes among the participants.
Caproni et al. (96)	2018	Breeding strategies for production of bean cultivars from landraces allowed the selection of suitable for organic farming.
Nadeem et al. (111)	2018	High genetic diversity was found in common bean from 19 different Turkish geographic regions.
Doma et al. (97)	2019	Older adults from north America considered beans as a healthy food that could improve their health.
Shi et al. (108)	2018	In different Canadian legumes, soaking decreased lectins and oxalates but not phytic acid. Cooking reduced all factors except phytic acid in beans and soybean.
Sparvoli et al. (109)	2021	Biofortified food products with common bean flour devoid of active lectins and with reduced content of phytic acid lost the haemagglutinating activity, increased α-amylase inhibitory activity and iron bioavailability.
Antoine et al. (110)	2021	In different meals, chickpeas reduced vitamin D and mineral transfer to the aqueous phase during digestion. The presence of meat induced a decrease in vitamin D stability.
Ferreira et al. (112)	2014	In Jalo and black beans species thermal treatment did not affect Cu, Fe, S, and Zn, but it increased Ca, K, Mg, P, and Zn concentrations.
Anene et al. (113)	2016	In mung bean seeds germination increased some macro-nutrients content while decreasing the anti-nutritional factors
Vaiknoras and Larochelle (114)	2021	Ten biofortified high-iron bean varieties showed higher yield than local varieties for improved household nutrition in Rwanda.
Sparvoli et al. (115)	2016	Several biscuit formulations containing lectin-free bean flour have a better amino acid score, higher fiber, glycemic index and starch content.

Common beans also contain numerous bioactive compounds, such as polyphenols, flavonoids, anthocyanins and carotenoids with different biological function (101). Phenolic compounds are thereby one of the most important families of phytochemicals present in beans with antioxidant and anti-inflammatory effects. They promote several benefits including reduction in the incidence of cancer, diabetes, cardiovascular diseases, and obesity. Furthermore, phenolic acids and flavan-3-ol reduce the risk of diseases in the digestive tract (102, 103).

Common bean seeds have 4–10 times more iron (Fe) than cereals such as maize, wheat and rice (104, 116). Significant variations in the iron content of staple crops exists, which has been linked to soil iron availability (Table 2) (105). In addition, Hummel et al. (100) reported that iron levels in common bean are reduced under relevant drought stress conditions, and consistent effects have recently reviewed by Losa et al. (117). In this regard, an increased research interest is in studying the bioavailability of Fe and how it is affected by food preparation approaches (106). For this issue, the International Center for Tropical Agriculture (CIAT), in consultations with nutritionists, established a breeding goal level of 94 mg/kg Fe above the value of a standard local variety to achieve 30% of average daily Fe requirement, assuming 7% bioavailability, 90% retention after cooking, and a high level of consumption of 200 g/day beans for adults and 100 g/day for children (118). In common beans, as in all plant foods, iron is found in non-haeme form, which has a lower bioavailability than haeme iron present in meat and fish. In turn, non-haeme iron can accumulate in different chemical forms, which significantly affects its absorption. Over 90% of the iron present in legumes is stored in the form of ferritin which positively affect bioavailability (Table 2) (107).

Although consumption of common bean is widely recommended for their health-promoting nutritional quality, their use, as well as other legumes, is limited by the presence of several antinutritional factors such as phytate, raffinose family oligosaccharides, trypsin inhibitors, lectins and tannins (108, 119, 120). Phytate, the salt of phytic acid, is widely distributed in the plant kingdom serving as a storage form of phosphors and minerals (121). Phytic acid acts as a strong cation chelator, reducing the bioavailability of important minerals such as iron, zinc, potassium, calcium, and magnesium (109, 122). Phytic acid can also impact on the bio-accessibility (i.e., the transfer to mixed micelles during digestion) of fat-soluble micronutrient such as vitamin D (Table 2) (110). Similar to trypsin inhibitors, lectins and tannins reduce digestion and absorption of dietary proteins by the formation of complexes that are resistant to digestive enzymes (123–126). Tannins also highly impact pancreatic lipase activity, which partly explains their negative impact on both vitamin D (110) and potassium bio-accessibility. Decreasing nutrient bioavailability, anti-nutrients have significant adverse effects on the nutritional value of foods and can become toxic when present beyond a certain amount. Therefore, reducing their concentration in foods is a major goal in human nutrition and suitable processing of these products, including legumes, it’s necessary before their consumption (127). For this purpose, numerous traditional methods and innovative techniques have been developed over the years to reduce the amount of antinutrients and improve the nutritional characteristic of legumes.

### 4.3 Strategies for improving nutritional quality of beans

Several traditional household food-processing, preparation and cooking methods are currently applied to enhance the bioavailability of micronutrients in plant food (86). In legumes, thermal processing, soaking, puffing, milling, fermentation, and germination/malting increases the physicochemical accessibility of micronutrients, decreasing the content of antinutrients, such as phytate, or increasing the compounds that improve bioavailability (Table 2) (112, 127, 128). For example, germination and malting are the most common legume processing methods, which are responsible for increasing iron absorption (129). This is because they help to improve the content of vitamin C or decrease the level of phytic acid, while sprouting enhances the bio-accessibility of Fe due to a reduction in the tannin content (86). However, previous results showed that reducing anti-nutrient content of legumes may be insufficient to have a significant impact on fat-soluble micronutrient bio-accessibility (110).

Various genetic tools are also currently applied to increase the production and utilization of micronutrient rich foods. These tools allow to develop bean varieties with an increased density of minerals, but also decreased amounts of inhibitors, or better food absorption (109, 113). Such genetic improvement of the nutritional value can change the anti-nutritional factors in legumes. In recent decades, research mainly focused on developing approaches aimed at reducing these antinutrients to further improve important nutritional properties to sensitize consumers for their regular consumption (109). Varieties of leguminous crops with high protein content and no anti-nutritional elements have been so far already developed, which resulted, for example, in products with a better protein availability (7).

Biofortification is further a process of increasing the density and bioavailability of vitamins and minerals in a crop through plant breeding, transgenic techniques, or agronomic practices. Biofortified staple crops are a viable means of reaching rural populations who may have limited access to adequate diets or other micronutrient-enhancing interventions (130). Conventional farming can be used to ensure a greater supply of vitamins or minerals to poor populations who depend on the specific staple crop. For example, biofortification of food crops for higher micronutrient levels of Fe, zinc (Zn) and provitamin A can boost these essential nutrients in the food system of targeted regions such as East Africa, South Asia and Latin America (131). Such breeding interventions have also been applied to develop high-yield legumes with a high micronutrient content, such as Fe biofortified beans. They are designed to address Fe deficiency, one of the most common micronutrient deficiencies globally (114) which causes anemia, fatigue, increased risk of infection, and pregnancy complications (130). Unfortunately, plant-based diets found in developing countries have low Fe bioavailability containing almost exclusively non-haeme iron, often absorbed less than 10% respect to haeme iron (113, 132). Anyway, the absorption of Fe does not only depend on its form, but also on the state of Fe in the organism: in case of deficiency, its absorption-in both forms-increases (132, 133). Furthermore, the bioavailability of Fe is influenced by the presence of its inhibitors, mainly phytates, polyphenols, calcium, milk and egg proteins, and by its enhancers such as ascorbic acid. It is, therefore, clear that the diet as a whole, influences the risk of anemia (56, 133). In addition, in common beans and legumes non-heme Fe in the form of ferritin ensures protection from the chelating action of phytic acid, making it more bioavailable. This effect is maintained during cooking and digestion, because phytoferritin remains intact during these processes (56).

In rural populations, biofortified beans can improve nutrition through two main pathways, primarily in the households that produce and consume them. The first is to increase nutrient intake through increased consumption of the home-grown biofortified crop, which has a higher iron content than other available bean varieties. The second path occurs by increasing the family income available for the purchase of other nutritious foods (120). Recent studies have been, therefore, conducted for the successful breeding of common beans using classical breeding methods to accomplish a wide array of objectives. This includes increasing the adaptation of the beans to different environmental conditions, developing genetically improved cultivars and increasing resistance against various biotic and abiotic stresses (96, 111).

#### 4.3.1 Current developments for the improvement of nutritional quality

All the approaches applied in the reduction of anti-nutritional dietary factors, allow the consumption and the use of legumes in the production of nutritionally balanced foods, which alleviate the challenge of protein and energy malnutrition in developing countries (7). In this way, the connection of conventional breeding and metabolic engineering strategies for combining nutritional and agronomic traits in the same genetic locus, can lead to large economic and social benefits in developing countries (134). Similarly, the improvement of the nutritional quality of beans contributes to the gradual transition from animal to plant-based protein food in middle and high-income countries, desirable to maintain environmental stability, ethical reasons, food affordability and fulfilling consumer demand (134, 135). From this perspective, the promotion of the consumption of legumes in these countries should also include the use of beans with improved nutritional characteristics as an ingredient in traditional recipes and as a component of products that meet market needs. Moreover, these improved common bean materials, particularly lines devoid of lectins, can also be exploited as ingredients in baked product preparations avoiding any previous flour processing (Table 2) (109, 115). Snacks obtained with common bean flour from these genotypes are also more protein rich than snacks from traditional flour (109, 115).

## 5 Conclusion

Raising awareness of the benefits of alternatives to the consumption of products of animal origin and the need to develop food chains that are part of a sustainable system for the environment and health, is essential to address in the future inequalities and achieve the multiple objectives of the 2030 Agenda (136).

In the context of the transition towards plant-based consumption patterns, research is underway to the impact on plant proteins on healthy diets and some results are critical if the evaluation is made at the level of nutritional adequacy. Authors found that plant-based meat substitutes can be levers for healthy diets only when well nutritionally designed with enough zinc and iron for a substantial red meat reduction. Plant-protein diversity was found positively associated with nutritional quality and more diverse plant protein intake, with higher contributions from legumes, nuts, seeds, and vegetables, appears to be critical in the context of increasing plant-protein (137, 138). Therefore, legumes including common beans are among the traditional protein sources that are and will still playing a central role in food and nutritional security, due to their characteristics related to diffusion, growth, variability, nutritional value and low production cost. They represent an excellent solution to the challenge of providing high quality dietary protein and micronutrients to the growing world population. The variety and versatility of legumes, especially beans, allows them further to be included in a vast range of dishes, as traditional grain-legume recipes, or new or “reinvented” products. They promote the diffusion of foods rich in fiber and protein, low in fat and that contribute to food security, sustainable agriculture and adaptation to climate change. Despite these advantages, however, production and consumption of legumes is still limited and urgently needs to be promoted. Challenges, however, concern not only more research in breeding intervention, for example metabolic engineering for substantially increase legume yield to provide more food for humans, but also exploring in much more depth the versatility of legumes as a key strategy to increase consumption and improve the transition to healthier sustainable diets. Biofortification, in combination with dietary diversification and nutrition education, holds great potential to eradicate micronutrient malnutrition and increase global human health.

## Author contributions

SL: Writing – original draft, Writing – review & editing. SM: Writing – original draft. CL: Writing – review & editing. ECa: Writing – review & editing. AA: Writing – review & editing. PG: Writing – review & editing. LG: Writing – review & editing. KK: Writing – original draft. DM: Writing – review & editing. BV: Writing – review & editing. KA-R: Writing – review & editing. ER: Writing – review & editing. ECo: Writing – review & editing. CP: Writing – review & editing. FS: Writing – review & editing. AL: Writing – review & editing. TS: Writing – review & editing. A-MB: Writing – review & editing. MF: Conceptualization, Supervision, Writing – original draft.
