# Supplementary figures and images for: Legumes and common beans in sustainable diets: nutritional quality, environmental benefits, spread and use in food preparations
Source: Front Nutr. 2024 May 6;11:1385232. doi: 10.3389/fnut.2024.1385232 (PMC11104268; doi:10.3389/fnut.2024.1385232)

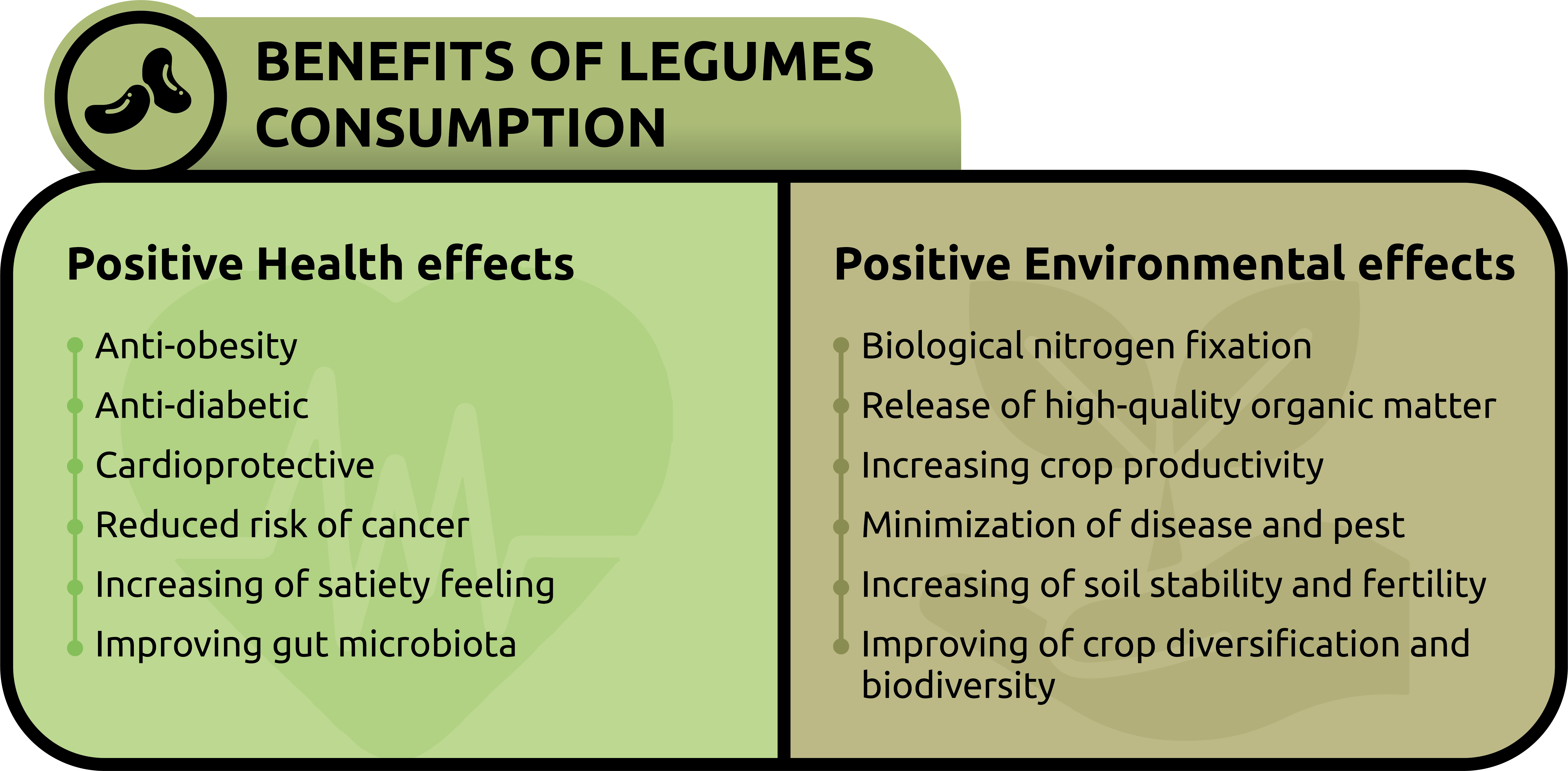

Supplement: Supplementary file 1 [file Image_1.PNG]
